# The Effect of Wall Material Type on the Encapsulation Efficiency and Oxidative Stability of Fish Oils

**DOI:** 10.3390/molecules26206109

**Published:** 2021-10-10

**Authors:** Khaled A. Selim, Salman S. Alharthi, Abdelmonam M. Abu El-Hassan, Nady A. Elneairy, Laila A. Rabee, Adel G. Abdel-Razek

**Affiliations:** 1 Department of Food Science and Technology, Faculty of Agriculture, Fayoum University, Fayoum 6351, Egypt; ama21@fayoum.edu.eg (A.M.A.E.-H.); nan00@fayoum.edu.eg (N.A.E.); lar00@fayoum.edu.eg (L.A.R.); 2 Department of Chemistry, College of Science, Taif University, P.O. Box 11099, Taif 21944, Saudi Arabia; s.a.alharthi@tu.edu.sa; 3 Department of Fats and Oils, National Research Centre, Dokki 12622, Cairo, Egypt; adelgabr2@gmail.com

**Keywords:** fish oil, oxidative stability, omega-3, encapsulation, spray drying

## Abstract

Fish oil is the primary source of long-chain omega-3 fatty acids, which are important nutrients that assist in the prevention and treatment of heart disease and have many health benefits. It also contains vitamins that are lipid-soluble, such as vitamins A and D. This work aimed to determine how the wall material composition influenced the encapsulation efficiency and oxidative stability of omega fish oils in spray-dried microcapsules. In this study, mackerel, sardine waste oil, and sand smelt fish oil were encapsulated in three different wall materials (whey protein, gum Arabic (AG), and maltodextrin) by conventional spray-drying. The effect of the different wall materials on the encapsulation efficiency (EE), flowability, and oxidative stability of encapsulated oils during storage at 4 °C was investigated. All three encapsulating agents provided a highly protective effect against the oxidative deterioration of the encapsulated oils. Whey protein was found to be the most effective encapsulated agent comparing to gum Arabic and maltodextrin. The results indicated that whey protein recorded the highest encapsulation efficiency compared to the gum Arabic and maltodextrin in all encapsulated samples with EE of 71.71%, 68.61%, and 64.71% for sand smelt, mackerel, and sardine oil, respectively. Unencapsulated fish oil samples (control) recorded peroxide values (PV) of 33.19, 40.64, and 47.76 meq/kg oil for sand smelt, mackerel, and sardine oils after 35 days of storage, while all the encapsulated samples showed PV less than 10 in the same storage period. It could be concluded that all the encapsulating agents provided a protective effect to the encapsulated fish oil and elongated the shelf life of it comparing to the untreated oil sample (control). The results suggest that encapsulation of fish oil is beneficial for its oxidative stability and its uses in the production of functional foods.

## 1. Introduction

Large quantities of fish waste (20–50% of the total fish weight) are produced during fish processing. Fish wastes are a huge amount, perishable, and are considered a threat to the environment when it is improperly disposed of and have no commercial value [1]. Many studies have been conducted regarding the production and characterization of fish oil as a by-product of fish processing. It has been illustrated that fish wastes are increasingly and being utilized to produce high-value products with functional and bioactive properties such as gelatin, protein hydrolysate, and omega-3 fatty acids concentrate [2]. Fish oil has many health and nutritional benefits due to its content of long-chain omega-3 fatty acids (n-3 PUFA), especially eicosapentaenoic (EPA) and docosahexaenoic (DHA), which are known for their health benefits [3,4]. Studies have shown that omega-3 fatty acids (EPA and DHA) can help develop the sense of sight, the immune system, and the brain development process (intelligence) in children [5]. The fatty acids EPA and DHA have also been found to be beneficial in lowering blood cholesterol, especially low density lipoprotin (LDL), and acts as an anti-platelet aggregation and anti-inflammatory [6]. However, one of the most important obstacles facing the use of oils containing a high amount of n-3 PUFA, such as fish oil (FO). It is prone to rapid oxidation due to the many double bonds found in the fatty acid molecule. The unpleasant smell of fish oil originating from this oxidation helps to evaluate the sensory characteristics of the product [7]. The encapsulation of sensitive or active components such as fish oil has become a very attractive process in recent decades. The utilization of liquid fish oil instead of hydrogenated oil among food manufacturers has also been increased. The microencapsulation technique seems to be a promising way of incorporating the oil into food products without sacrificing the organoleptic properties [8].

The microencapsulation process can be defined as the coating technique of small solids, gases, and liquids capsules. It protects the encapsulated materials against oxidation, improving the oil stability and prolonging its shelf life, and releases it at specific rates under controlled conditions [9,10]. Spray drying as a microencapsulation technique is most commonly used for commercial production and heat-sensitive materials because of its relatively short drying time, low cost, and ability to prepare microcapsules with high quality [11,12].

Many encapsulating agents have been employed as wall materials on fish oil encapsulation. Among these agents, sugar beet pectin, gum Arabic, gelatin, maltodextrin, skimmed milk, starch, chitosan, and whey proteins [13,14]. According to our information, most of the fish oil in the market is produced from the body of fish in addition to cod liver oil, and this increases the cost of production. Therefore, the use of fish processing by-products reduces the cost of production and increases the benefit from these by-products, and the production of fish oil in powder form makes it available for use in the food industry. The objective of this work is to produce a dried fish oil protected from oxidation and make sure the fish flavor is relatively masked by means of encapsulation using different encapsulating agents (whey protein, maltodextrin, and gum Arabic) and to investigate the effects of these agents on the encapsulation efficiency and oxidative stability of fish oils in spray-dried microcapsules.

## 2. Results and Discussion

### 2.1. Physiochemical Characteristics of Extracted Fish Oils

**Figure 1:**
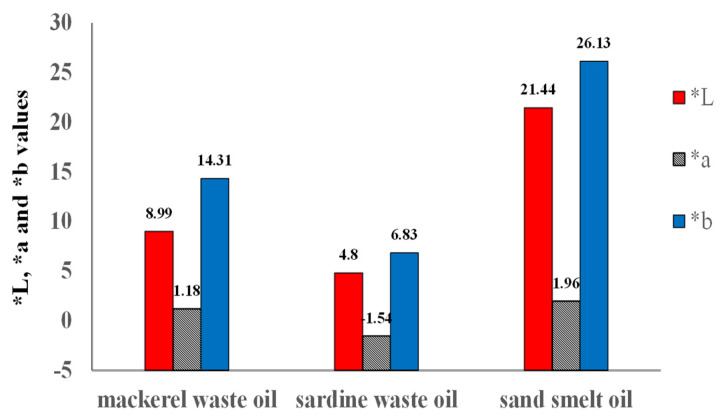
Color parameters *L, *a, and *b values of oil samples.

The by-products of fish manufacturing include the tail, head, intestines, fins, skin, and skeleton, which can be used in the production of many nutritional value products such as proteins, oil, collagen, and gelatin [15]. There were significant differences between the investigated oils in the crude lipid content. Mackerel waste recorded the highest crude lipids, while sand smelt fish had the lowest crude lipids content. On the other hand, mackerel waste oil recorded flow time lower than those of sardine waste oil and sand smelt fish oil (Table 1). These values were lower than those reported by Sellami et al. [15]. Specific gravity values were (0.912 ± 0.002, 0.947 ± 0.022, and 0.928 ± 0.003) for mackerel and sardine waste oils and sand smelt fish oil, respectively. The highest acid value was obtained from sardine fish waste oil, followed by mackerel waste oil. High acid values in fish waste oils could be due to fish oil being highly prone to both lipolysis and oxidation. These results are in agreement with those reported by Aryee et al. [16]. The acceptable limit for the acid value of crude fish oil is 2–5. Peroxide values (PVs) of all examined samples were in the acceptable range. However, according to Boran et al. [17], the acceptable limit for PV of crude fish oil is 2–20 meq peroxide/kg oil. All examined samples did not exceed this limit. Peroxide values examined oil ranged between (from 2.28 ± 0.04 to 2.67 ± 0.11 meq/kg oil). These results are in agreement with those reported by Smichi et al. [18], who found that the PVs were 1.2, 1.6, and 2.6 meq/kg for the freshly extracted oils from annular sea bream, sardine, and golden grey mullet, respectively. Color in sardine and mackerel waste oils and sand smelt fish oil are given in Table 1 and Figure 1. The results showed significant differences between L* values and b* values for all the tested oil samples. The highest L* (lightness) value of 21.44 ± 0.16 was found in sand smelt fish oil. However, mackerel and sardine showed lower L* values of 8.99 ± 0.007 and 4.80 ± 0.08, respectively. The positive value of a* gives an indication of the redness fraction of the extracted oil’s color. The a* (redness) value was higher (1.18–1.96) in mackerel waste oil and sand smelt fish oil than that of sardine waste oil (−1.54 ± 0.13). The b* value is a measure of yellowness and was observed to be highest in sand smelt fish oil and mackerel waste oil 26.13–14.31 and the lowest b* value 6.83 ± 0.06 was shown in sardine waste oil. These results are in agreement with those found by Tomoko et al. [19]. These results could be attributed to the carotenoids pigments in extraction higher in the mackerel waste oil and sand smelt fish oil.

Similar refractive index values were found for all investigated oils. The refractive index recorded values of 1.479 ± 0.0021, 1.480 ± 0.01, and 1.470 ± 0.001 for mackerel and sardine waste oils and sand smelt fish oil, respectively. Regarding the flow time, mackerel waste oil showed the lowest flow time (5.70 s). These values were lower than those reported for the liver oil of three ray species [15]. Specific gravity can be used to determine the purity of oil to match desired standards. The specific gravity of sardine, mackerel, and sand smelt fish oil is shown in Table 1; the presented results indicated that specific gravity was (0.912 ± 0.002, 0.947 ± 0.022, and 0.928 ± 0.003) for mackerel, sardine, and sand smelt fish oil, respectively. The highest value of specific gravity was observed in sardine, and the lowest value was recorded for mackerel waste oil.

Fish cannot synthesize carotenoids, so they obtian them from food that contains them, such as algae, small crustaceans, and phytoplankton in the water [20,21]. Due to their lipid-soluble nature, carotenoids are extracted with oil. Therefore, this study has determined the carotenoid concentrations in oils extracted from the fish and fish waste oils (Table 1). Carotenoid contents as astaxanthin in the extracted oils varied significantly (*p* < 0.05) among the studied oils, with values ranging from 128.34 ± 3.78 µg/g for mackerel waste oil to 98.12 ± 1.56 µg/g for sardine waste oil. Carotenoids content of the oil could affect the stability of the encapsulated oils because of its antioxidant activity. This may explain the higher peroxide value in sardine oil compared to other oils due to its lower carotenoids content (98.12 ± 1.56 µg/g). Our results are higher than those reported by Sellami et al. [15] for the liver oils from ray species living along Tunisian coasts.

### 2.2. Fatty Acid Composition (%) of Extracted Oils

Fatty acids compositions of the studied fish wastes oils are shown in Table 2. The fatty acids of the oils extracted from sardine and mackerel waste oils and sand smelt fish oil are composed of saturated fatty acid, monounsaturated fatty acid, and polyunsaturated fatty acid. The fatty acid profiles exhibited a dominance of unsaturated fatty acids (UFAs) for all samples between 55.5 and 78.86% of the total fatty acid content. These findings are in line with those previously reported by Negre-Sadargues et al. and Cynthia et al. [21,22]. The total saturated fatty acids (SFA) content of fish and fish waste oils ranged between 7.16 and 20.80%, which was lower in sardine waste oil and higher in sand smelt fish oil. The predominant SFAs in sardine waste oil and sand smelt fish oil were stearic acid. The highest levels of stearic C18:0 (11.9%) were recorded in sand smelt fish oil.

The total monounsaturated fatty acid (MUFA) of the investigated oil samples were found to be 4.04, 29.13, and 47.79 for sardine, mackerel, and sand smelt fish oils, respectively. The main MUFA identified was oleic acid C18:1 in sand smelt fish oil in this study; oleic acid (18:1 *cis* ω-9) was the most represented of the total MUFAs in sand smelt fish oil (30.59% of total FAs) and mackerel waste oils (11.65% of total FAs). Similar findings for Kilka fish oil were reported by Hosseini et al. [23].

The total polyunsaturated fatty acids of sardine, mackerel waste oils, and sand smelt fish oil were determined to be 51.46%, 43.09%, and 31.07%, respectively. The highest levels of PUFAs were observed for sardine waste oil of 51.46%. The major n-3 PUFAs were EPA (C20:5) and DHA (C22:6). Eicosapentaenoic acid (EPA) fatty acids in sardine, mackerel waste oils, and sand smelt fish oil were 23.27, 18.63, and 1.7%, respectively, while docosahexaenoic acid (DHA) content of the examined oils ranged from 16.27 to 5.40%. For PUFAs, the major fatty acids identified were eicosapentaenoic acid (EPA, C20:5 ω-3) and docosahexaenoic acid (DHA, C22:6 ω-3). The highest EPA values were obtained in sardine waste oil and mackerel waste oil (23.27 and 18.63% of total fatty acids (TFA), respectively). The highest rates of DHA were found in sardine and mackerel waste oils, 16.27 and 15.49% of TFA, whereas sand smelt fish oil was found to have 1.7% EPA and 5.4% DHA of TFA; our findings are symmetrical with these reported by Cynthia et al. [20] and higher than those reported by Hosseini et al. [23] for EPA and DHA content in sardine waste oil (3 and 6.23%, respectively). The composition is similarly shown by other fish lipids, such as salmon and rainbow trout [24]. On the other hand, the results of fatty acid composition of sardine waste oil are dispute by those obtained by Benguendouz et al. [25], who reported that the fatty acid compositions of sardine fillets oil ranged between 35.50% and 41.32% for saturated (SFA), 14.22–22.27% for monounsaturated (MUFAs), and 36.63–47.96% for polyunsaturated acids (PUFAs). Comparatively, the results which expressed the different proportion of MUFAs and PUFAs with other fish lipids may be due to the environmental effect of tropical fish species [26]. The distinctive difference of the SFAs, MUFAs, and PUFAs content in sardine waste oil and other fish lipids may be attributed to the seasonal changes and the changes in plankton species in their diet and in the post-spawning period [27].

The variations of n-3 PUFA of sand smelt fish oil and other fish waste oils are shown in Table 2. The results showed that the n-3 PUFA content of sardine waste lipid was higher than that of mackerel waste oils and sand smelt oil compared to some other researches on *Sardina pilchardus* with C22:6 n-3 levels of 11.30% [28].

### 2.3. Physical Properties of Encapsulated Fish Oils

#### 2.3.1. Moisture Content of Encapsulated Fish Oils

The moisture content of the sardine, mackerel waste oils, and sand smelt oil powders obtained from the spray-drier process is given in Table 3. The moisture content of the encapsulated oils was between 1.93% and 3.51%. The maximum moisture content of encapsulated oils was recorded for sardine waste oil encapsulated in gum Arabic of (3.51%), while mackerel waste oil encapsulated in whey protein showed the lowest moisture content of 1.93%. The moisture content of different formulations was compared and found to be statistically significant (*p* ≤ 0.05). These results are in agreement with the previous findings reported by [29,30]. Moisture content could act as an antioxidant at very low levels by decreasing the catalytic activity of metal catalysis and reducing free-radical formation. When the water content is increased, the oxidation rate increases by increasing the mobilization of the reacting compounds. It appears to have a plasticizing effect in the matrix and makes it vulnerable to many reactions that accelerate the oxidation process. The results indicated that oils encapsulated in whey protein recorded the lowest moisture content, which led to lowering the oxidation rate during storage [31]. The low moisture content is one of the important factors that maintain the quality of the oil and lead to prolonging its shelf life by reducing the oxidation rate. Rahmani-Manglano et al. [31] reported moisture contents of 4.7% ± 0.4% and 4.2% ± 0.1% for fish oil encapsulated in maltodextrin (DE 21) and glucose syrup, respectively. It was reported that the maximum moisture specification for most dried powders in the food industry is between 3% and 4%.

#### 2.3.2. Encapsulation Efficiency of Encapsulated Fish Oil Powders

Encapsulation efficiency was calculated based on total oil content and surface oil content, and the results are presented in Table 3. The results showed that the encapsulation efficiency of the encapsulated fish and fish waste oils powder varied from 45.55% in MD + SSO to 71.16% in WP + MAO. When maltodextrin was used alone, it showed EE ranged between 45.55 and 57.25%. Further, higher EE 71.16% was observed for mackerel waste oil encapsulated in whey protein as a wall material, which means that a large amount of the oil was encapsulated within the carrier used, and less was adsorbed onto the surface of the microcapsules. Several studies have indicated that the type of carrier used and its chemical properties, in addition to the properties of the resulting emulsion, have a significant impact on the stability of the microencapsulated materials [32,33]. The results indicated that whey protein showed the highest encapsulation efficiency compared with gum Arabic and maltodextrin in all encapsulated samples. It has been reported that the use of whey protein as a wall material was able to provide good encapsulating properties. The obtained results are in agreement with those reported by Hogan et al. [32]. An encapsulation efficiency of 46.83–49.34% for ginger essential oil encapsulated in milk protein and fish gelatin was reported by Jeyakumari et al. [33]. In another study, Binsi et al. observed an encapsulation efficiency of 68.99–73.21% for sage polyphenols is encapsulated in sodium caseinate and gum Arabic [34].

#### 2.3.3. The Flowability of Powders

The flowability of the powder is one of the important properties that affect the handling of the powder and the extent of its transportability and storage, as well as the ability of the powder to be mixed, processed, and packaged [35]. The higher Carr′s index and Hausner ratio means that the powder is more cohesive and less able to flow freely [36]. In the present study, higher Carr index (27.66, 26.51, and 30.19) and Hausner ratio (1.38, 1.36, and 1.43) was observed for sand smelt fish, mackerel, and sardine fish waste oils encapsulated in maltodextrin, respectively, which indicated poor flowability of the spray-dried powder (Table 4). These results are in agreement with those reported by Turchiuli et al. [37].

On the other hand, a low Carr index (17.51, 16.24, and 18.64) and Hausner ratio (1.21, 1.19, and 1.23) was observed for fish oil and fish waste oils encapsulated in whey protein as a wall material, which indicated fair flowability of the spray-dried powders.

### 2.4. Oxidative Stability of Encapsulated Fish Oils

#### 2.4.1. Peroxide Values (PV) of Encapsulated Fish Oils during Storage at 4 °C for 2 Months

**Figure 2:**
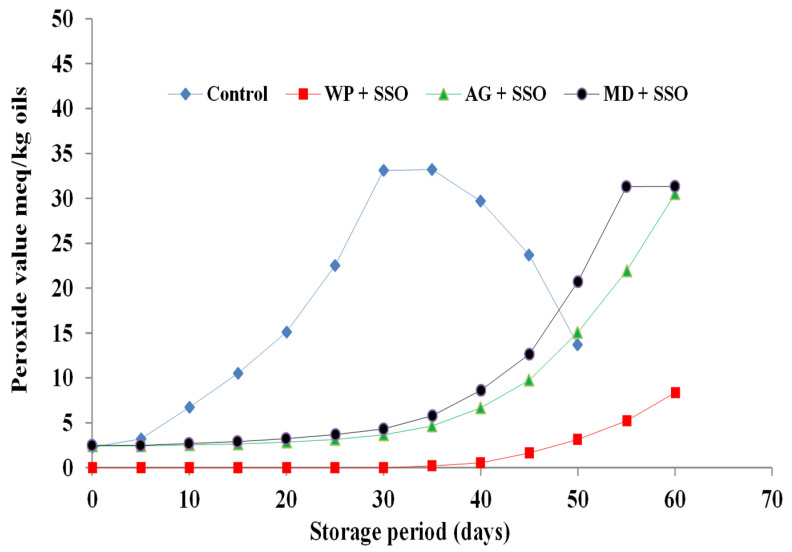
Peroxide value of encapsulated sardine waste oil during storage at 4 ± 0.5 °C.

**Figure 3:**
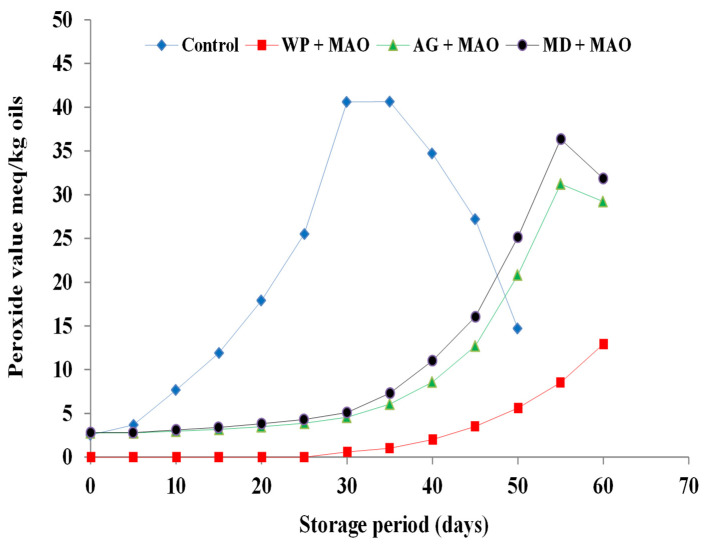
Peroxide value of encapsulated mackerel waste oil during storage at 4 ± 0.5 °C.

**Figure 4:**
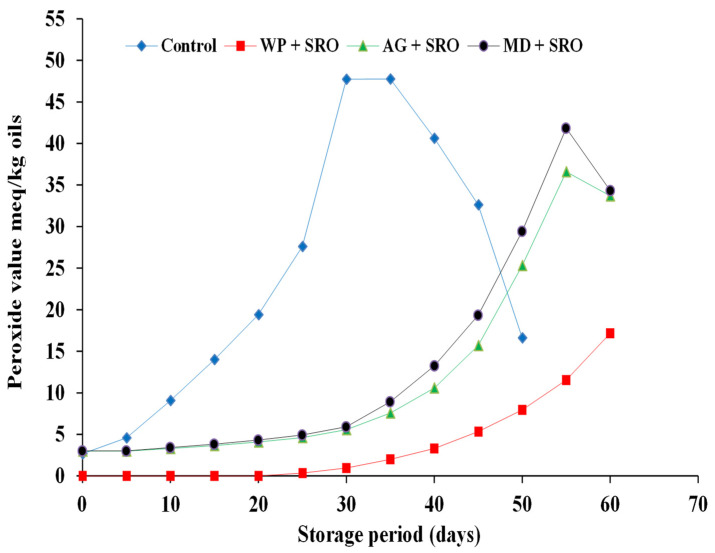
Peroxide value of encapsulated sand smelt fish oil during storage at 4 ± 0.5 °C.

The initial PVs of fish oils encapsulated in whey protein were zero for sardine, mackerel waste oils, and sand smelt fish oil, while it was 3.02, 2.81, and 2.47 meq/kg oil in freshly prepared encapsulated sardine and mackerel waste oils and sand smelt fish oil that contained maltodextrin as a wall material. This observation was obvious as the emulsion droplets were temporarily exposed to air and high temperature during spray drying [38]. The PV of un-encapsulated sardine and mackerel waste oils and sand smelt fish oil increased rapidly during storage and reached 47.76, 40.64, and 33.19 meq/kg, respectively, after 35 days of storage. In that same period, the PV of encapsulated sardine and mackerel waste oils and sand smelt fish oil using whey protein, gum Arabic, and maltodextrin as a wall material increased slowly (Figure 2, Figure 3 and Figure 4).

Peroxide values of encapsulated fish and fish waste oils using whey protein as a wall material ranged from 0 to 16.44, from 0 to 12.92, and from 0 to 8.33 meq/kg for encapsulated sardine and mackerel waste oils and sand smelt fish oil, respectively. These results could also be attributed to the physicochemical properties of the whey protein, which provide excellent emulsification, film-forming capabilities, and a high ability to self-associate into networks. The protection effect offered by a matrix is depended on its chemical characteristics, the physical state, and the macrostructure of encapsulating agent (i.e., the extent of its surface, the size, shape, and distribution of pores). On the other hand, the results showed that encapsulated sardine waste oil using whey protein as a wall material has the highest PV compared to mackerel waste oil and sand smelt fish oil. This means that the oxidative stability of sardine waste oil is lower than sand smelt fish oil and mackerel waste oil. The results also observed that the PV of fish and fish waste oils encapsulated in whey protein as a wall material increased from 0 to 1.63, 3.52, and 5.32 meq/kg oil in 45 days for encapsulated sand smelt fish oil and mackerel and sardine waste oil, respectively, whereas the PV of the samples encapsulated in gum Arabic and maltodextrin increased to 9.76, 12.68, and 15.69 and 12.62, 16.04, and 19.34 meq/kg oil, respectively (Figure 2, Figure 3 and Figure 4). The results showed that there were significant differences between the control samples and all the encapsulated fish and fish waste oils during storage. It also proved that there were significant differences (*p* ≤ 0.05) between the whey protein, gum Arabic, and maltodextrin as wall materials in protecting the encapsulated oils during storage. The results indicated that the highest PV was observed for un-encapsulated sardine waste oil after 35 days of storage (47.76 meq/kg oil) followed by mackerel waste oil (40.64 meq/kg oil), while sand smelt fish oil recorded the lowest PV at the same period of storage and recorded a PV of 33.19 meq/kg oil. These results indicate the influence of the wall material (e.g., which determines the permeability to prooxidant species and retention properties) on the oxidative stability of microencapsulated fish oil. It was observed that peroxide values of the microencapsulated oils were below 20 meq O_2_/kg in all the fish oil after 45 days of storage, which were comparable with the standard specification value of 20 meqO_2_/kg accepted in oils and fats. The results conclude that using whey protein as encapsulating agents proved the lowest PV in 45 days for all encapsulated samples. It is argued that the early formation of particle crust and the lower surface oil content could probably shield the oil from oxidation attacks. The results are in agreement with those reported previously [39,40,41].

#### 2.4.2. TBA Value of Encapsulated Fish Oils during Storage at 4 °C in the Refrigerator

**Figure 5:**
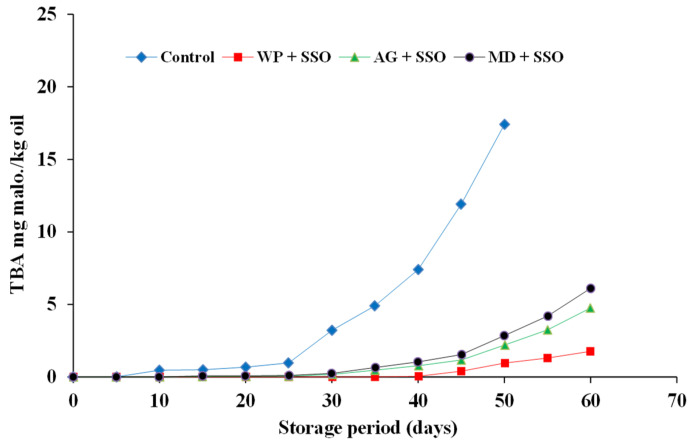
Thiobarbituric acid ( TBA)values of encapsulated sand smelt fish oil during storage at 4 ± 0.5 °C.

**Figure 6:**
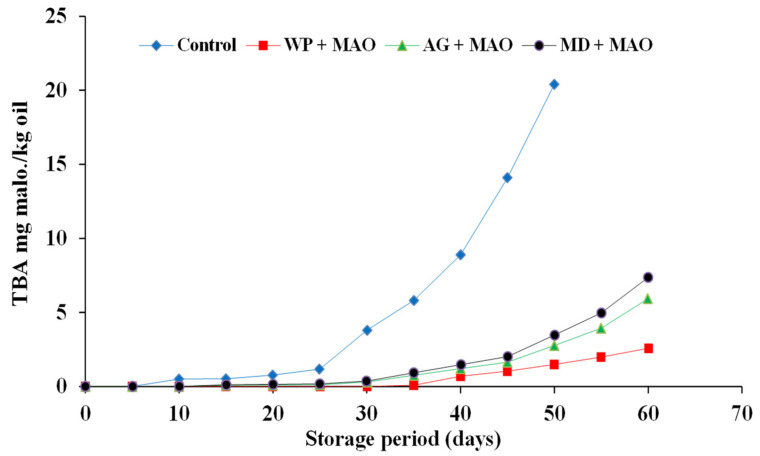
TBA values of encapsulated mackerel waste oil during storage at 4 ± 0.5 °C.

**Figure 7:**
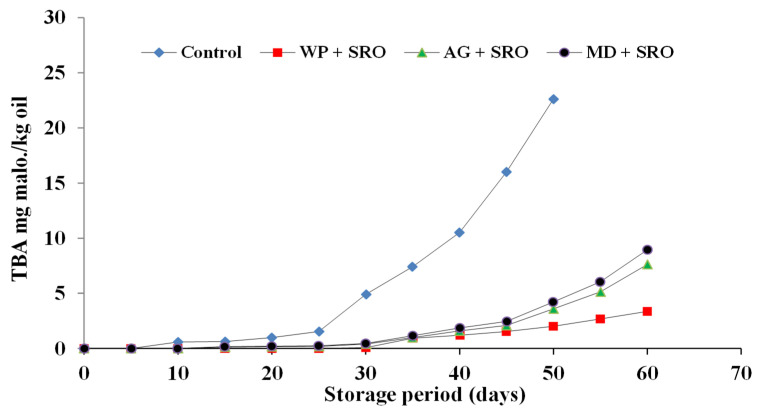
TBA values of encapsulated sardine waste oil during storage at 4 ± 0.5 °C.

The primary fat oxidation products are flavorless and colorless, so they cannot be distinguished by the consumer. Secondary fat oxidation products are among the products that have an odor, and therefore their assessment is important in the determination of fat oxidation in food products. Changes in the thiobarbituric acid (TBA) value of encapsulated fish oil during storage at 4 °C are shown in Figure 5, Figure 6 and Figure 7. A similar trend of peroxide values was also observed with respect to TBA values in the present study. The degree of oxidation (TBA values) was gradually increased in both fish and fish waste oils (control) and encapsulated fish and fish waste oils with the increasing of storage time. TBA values of sardine and mackerel waste oils and sand smelt fish oil were dramatically increased throughout storage time from (0 at zero time to 22.61, 20.4, and 17.41 mg malonaldehyde/kg oil after 50 days of storage), respectively. On the other hand, the increment of TBA values in encapsulated fish and fish waste oils was not so remarkable (0 as a minimum at zero time, growing to 4.21 for MD + SRO as maximum mg malonaldehyde/kg oil after 60 days of storage). It is clear that the encapsulation of fish oil strongly delayed the oxidation development in the powder.

The results showed that there were significant differences (*p* ≤ 0.05) between the control fish oils and the encapsulated fish oils during the storage time. The results also indicated that whey protein as an encapsulating agent was superior to the other carriers in protecting the encapsulated fish oil [42]. TBAR values were found to be lower in the microcapsules in comparison to the fish oil and the emulsion indicating the protective effect of microencapsulation against ω-3 fatty acid oxidation. The levels of secondary lipid oxidation products were lower in the fish oil encapsulates in whey protein when compared to the fish oil encapsulated in gum Arabic and maltodextrin. In the present study, initial TBA values corresponded to 0 mg malonaldehyde kg/oil encapsulated and showed an increasing trend during storage. Results indicated that the oils encapsulated in maltodextrin showed higher TBA values of 2.85, 3.47, and 4.21 mg malonaldehyde kg/oil for sand smelt oil and mackerel and sardine waste oils, respectively, after 50 days of storage, while the oils encapsulated in whey protein showed lower TBA values of 0.96, 1.48, and 2.01 mg malonaldehyde kg/oil for sand smelt oil and mackerel and sardine waste oil, respectively, after 50 days of storage. It could be indicated that after 60 days of storage, all the recorded The thiobarbituric acid reactive substances (TBAR) values were comparable with the standard TBAR values for oils and fats for the encapsulated oils using different encapsulating agents.

#### 2.4.3. Acid Value (AV) of Encapsulated Fish Oils during Storage at 4 ± 0.25 °C

**Figure 8:**
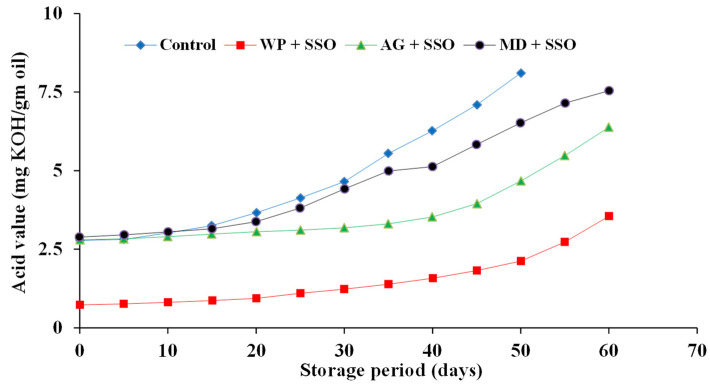
Acid value of encapsulated sand smelt oil during storage at 4 ± 0.5 °C.

**Figure 9:**
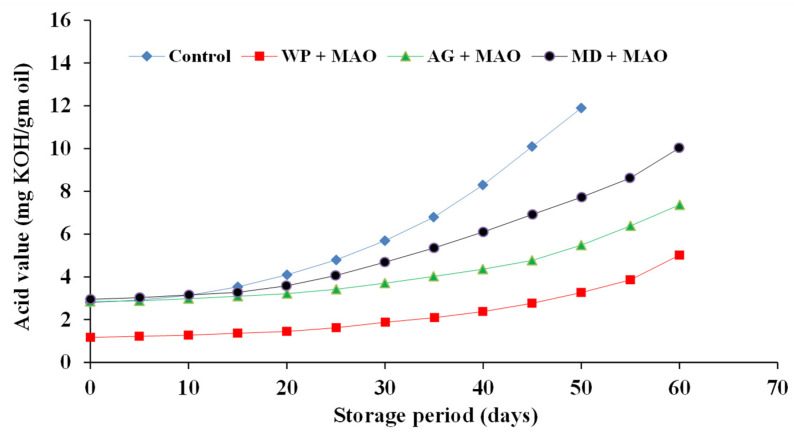
Acid value of encapsulated mackerel waste oil during storage at 4 ± 25 °C.

**Figure 10:**
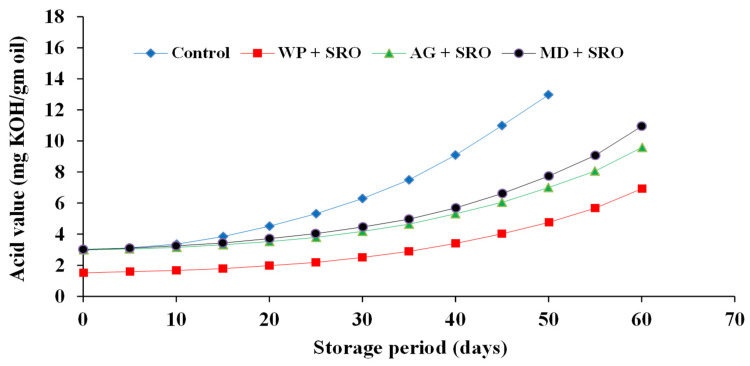
Acid value of encapsulated sardine waste oil during storage at 4 ± 0.25 °C.

The change of AV for the investigated oils is presented in Figure 8, Figure 9 and Figure 10. The results showed that there were significant differences between the controls and all tested samples. Regarding the type of oil, the results proved that sand smelt oil was the most stable during storage and recorded the lowest AV compared to the control and the encapsulated mackerel and sardine waste oils. It could be seen that encapsulated sardine waste oil showed the highest AV after 60 days of storage at 4 ± 0.25 °C and recorded an AV of 6.93, 9.58, and 10.95 for oil encapsulated in whey protein, gum Arabic, and maltodextrin, respectively. The results also showed that whey protein as an encapsulating agent was superior to gum Arabic and maltodextrin in all tested oils, and there were significant differences (*p* ≤ 0.05) between the samples encapsulated in whey protein and the other samples in the investigated oils. The oil samples encapsulated in whey protein recorded an AV of (3.55, 5.01, and 6.93) after 60 days of storage for sand smelt, mackerel, and sardine waste oils, respectively. These results are consistent with these reported by Matthew et al. [43].

## 3. Materials and Methods

### 3.1. Materials

Sand smelt fish and sardine and mackerel wastes were collected from markets and local fish restaurants, Fayoum Governorate, Egypt. Powdered maltodextrin (DE 15), gum Arabic and whey protein were purchased from Sigma Chemical Co. (St. Louis, MO, USA)

### 3.2. Methods

#### 3.2.1. Extraction of the Oil from Fish Wastes

Fish oils were extracted using direct steaming method according to Moorthy et al. [44]. After being thawed at ambient temperature, the fish wastes were placed in a muslin bag on a metal screen over an aluminum pan. The pan containing the fish waste was cooked by steam for 30 min. The fish waste in the muslin bag was then hand pressed and the oil and water released were combined with the fish waste during steaming. The fish waste in the muslin bag was hand-pressed, and the released emulsion of oil and water was combined with the fish waste during extraction with steam. The oil was separated from the emulsion by centrifuging at 2000 rpm for 15 min, dried using anhydrous sodium sulfate.

#### 3.2.2. Physicochemical Analysis of Extracted Oils

##### Physical Analysis of Extracted Oils

Specific gravity was determined according to the method described previously by the official methods of analysis (AOAC) using a 25 mL pycnometer. The results were standardized to 25 °C. A Carl Zeiss refractometer was used for measuring the refractive index for oils [45]. Flow time of the studied oils was measured as an index of viscosity according to the method reported by Fasina et al. [46].

##### Color Analysis of Extracted Oils

Color data were reported in CIELAB color scales *L, a*, and b* values of the extracted oils were determined according to the method reported by Anandan et al. [47].

##### Chemical Analysis of Extracted Oils

(a) Identification and determination of fatty acids in oils by gas chromatography (GC-MS).

Methyl esters of fatty acids were prepared in accordance with the method of A.O.C.S. [48]. For sample of 100 mg, 1 mL Boron triflouride BF3/methanol (14%) and 1 mL hexane is added. Then, the tube is vortexed and placed under nitrogen for 60 min at 100 °C. Esters of fatty acids were extracted by adding 1 mL of hexane and washing with 2 mL of distilled water. After the centrifugation step (4500 rpm, 10 min, 20 °C.), the supernatant is recovered in vials and then injected into the GC column (CG-2010 Plus, Shimadzu, Kyoto, Japan), equipped with a flame ionization detector and a capillary column of 60 m length, 0.25 mm internal diameter, and 0.20 µm film thickness. The oven temperature was 200 °C. The detector and the injector are at a temperature of 250 °C. The carrier gas was helium, the gas flow rate was 0.8 mL/min. The temperature program used in the analysis is to keep the unit at 120 °C for 2 min and then climb to180 °C for 2 min and keep the sample at 220 °C for 25 min. The peak integration is completed on the software GC solution (Shimadzu, Kyoto, Japan). Peak identification of fatty acids on the chromatogram is completed using standard fatty acids (Restek, Food Industry FAME Mix-methylene chloride 30 mg/mL).

(b) Peroxide values and acid values were estimated as described by A.O.C.S. [48].

For the peroxide values (PV), fish oil sample (5 g) was weighed into a 200 mL conical flask and mixed with 300 mL of glacial acetic acid and chloroform (3:1) and mixed thoroughly by swirling the flask. Saturated potassium iodide (0.5 mL) was then added, and the mixture was left in the dark for 1 min with occasional swirling, followed by further addition of 30 mL distilled water. The mixture was titrated with 0.1 N sodium thiosulphate solution with 1 mL of 1.0% soluble starch as indicator until the blue color disappeared. A blank sample titration was also carried out in the same manner but with no oil added.

(c) Thiobarbituric acid (TBA) value.

The oxidative level of fish oil and microcapsules was determined by the thiobarbituric acid reactive substance (TBAR) method, as described by Wan et al. [49]. Exactly 0.67 g of thiobarbituric acid was dissolved in distilled water with the aid of heat from a steam bath. The thiobarbituric acid solution was transferred to a 100 mL volumetric flask. It was cooled and made to volume with distilled water. An equal of the TBA solution was mixed with an equal volume of glacial acetic acid to prepare the TBA reagent. A known weight of the oil samples (3.0 g) were pipetted or weighed into a glass stopper red Maisel-Gerson tube. The sample was dissolved in 10 mL carbon tetrachloride. A total of 10 mL of the TBA reagent was then pipetted into the Maisel-Gerson tube and shacked in a horizontal position for a period of 4 min at approximately 125 oscillations per minute. The contents were then transferred to a separator funnel and the aqueous layer was withdrawn into a 25 × 200 mm test tube. The tube was then immersed in a boiling water bath for 30 min. Finally, absorbance at 532 nm was measured in a spectrophotometer Jenway 7305 (Roissy, France) using ternary solvent mixture as a blank. Malondialdehyde (MDA) equivalence was calculated from the calibration curve. Thiobarbituric acid reactive substances TBARS level was expressed as nmol Malondialdehyde MDA/kg oil.

##### Encapsulation of Extracted Fish Oils

(a) Preparation of emulsions

Preparation of emulsions was carried out as described by [50,51]. Hydrated solution of continuous emulsion phase was prepared by dissolving wall material powders in distilled water using a high-speed blender (Model RW 20.n, IKA Works, Kuala lumpur, Malaysia). For starch-based biopolymers, the temperature of water bath was adjusted to 60 °C, while for proteins, they were kept at ambient temperature to avoid changes due to temperature. In the case of whey protein concentrate (WPC), their solutions were prepared by dispersing the desired amount of their powder into buffer solution (5 mM phosphate buffer, pH 7). The pH of WPC solutions was adjusted back to pH 7.0 using 1 M HCl if required. The total concentration of dissolved solid was 30% (*w*/*w*) that was composed of 30 wt.% wall material and 10 wt.% of oil. All emulsions produced were of the oil-in-water type and were prepared in two stages: (a) pre-emulsions were obtained by a rotor–stator system (Model L2R, Silverson Machines Ltd., Chesham, UK). The Silverson is a typical colloid mill with a stator composed of a metal grating in which 2 mm holes are drilled. The core material (fish oil) in the ratio of 1:3 (core:wall) was progressively added to the continuous phase during pre-emulsion preparation and stirred for 10 min at the highest speed. (b) These coarse emulsions were then further emulsified using a Microfluidizer (Model M-110 L, Microfluidics, Storrs, CT, USA) at 60 MPa for one cycle or a 24 KHz Ultrasound probe (Dr. Hielscher series, Model UP 400S) with 22 mm diameter at the highest power for 100 s [50,51]. Sodium azide (0.02 wt.%) was added to the emulsions as an antimicrobial agent along with the addition of emulsifier agent (Span: Tween, 2:1). For each emulsifying device, about 1000 mL sample was prepared for the production of encapsulated powders by spray-drying.

(b) Spray-drying

The emulsions were spray-dried in a spray drier using air inlet temperature of 180 °C, air outlet temperature of 65 °C, and nozzle air pressure of 310 kPa. After drying, the powder was packed in dark-colored containers, tightly closed, and stored at 4 °C until analysis.

##### Moisture Content and Encapsulation Efficiency of Encapsulated Oils

(a) Moisture content

The moisture content of the microcapsules was determined according to the method reported by A.O.A.C. [45].

(b) Encapsulation efficiency (EE)

Encapsulation efficiency (EE) and powder flowability of encapsulated fish and fish waste oils were estimated according to the method depicted by Bae et al. [52].

##### Determination of Flow Properties of Encapsulated Oils

(a) Bulk Density (ρB)

Bulk density (ρB) was determined according to the method previously reported by Chinta et al. [53].

The volume (V_0_) was read directly from the cylinder and used to calculate the bulk density.

Bulk density (ρB) = m_0_/V_0_.

where m_0_ indicates the sample weight (g).

(b) The Tapped Density (ρT), Carr’s Index, Hausner Ratio, and Powder Flowability were determined and calculated as described by Chinta et al. and Turchiuli et al. [37,53].
(c) Powder flowability:

Powder flowability was calculated from the Carr’s index and Hausner ratio according to method followed by Turchiuli et al., [37] based on the Table 5 given below.

##### Determination of Oxidative Stability of Encapsulated Fish Oils

Oxidation degree of the encapsulated fish waste oil was followed periodically, as described by Hu et al. [54]. Along 60 days of storage at 4 ± 0.5 °C, degree of oxidation was determined in encapsulated oils every 5-day interval and expressed as peroxide value, thiobarbituric acid, and acid value and compared to the control samples, which contain oil only without any encapsulated agents.

(a) Thiobarbituric acid (TBA)

Thiobarbituric acid of the encapsulated fish waste oil was followed periodically, as reported by Wan et al. [49].

(b) Acid value (AV)

Acid values of encapsulated fish and fish waste oils were determined according to A.O.C.S. Recommended Practice Cd 1d-92 with modification. A total of 0.5 g of powder was dispersed in 10 mL of hexane and 10 mL of neutral alcohols and titrated by KOH with phenolphthalein as indicator [48].

#### 3.2.3. Statistical Analysis

The data were expressed as means of 3 replicates ± standard deviation. The data obtained were analyzed by one-way analysis of variance (ANOVA) using statistical analysis software SPSS version 19 (SPSS, Chicago, IL, USA). All mean separations were carried out by Duncan’s multiple range test using the significance level of 95% (*p* < 0.05).

## 4. Conclusions

The encapsulation of fish oils by spray-drying affected the efficiency and stability of fish oil. The encapsulation of fish waste oil improved the efficiency of fish oil with respect to the conventional stability of fish oil. Results obtained also showed that all the encapsulating agents used prolonged the shelf life of the encapsulated oils and made them more resistant against oxidative stress process. The whey protein increased the stability of fish waste oils against oxidative deterioration compared to fish waste oils encapsulated in gum Arabic, maltodextrin, and non-encapsulated fish oils. It may be generally applied to carry and deliver omega-3 fatty acids in powder form with extended shelf life and improved functional characteristics. To improve the shelf life, natural antioxidants could be added to the mixture.

## Figures and Tables

**Table 1:** Physicochemical properties of sardine and mackerel waste oils and sand smelt fish oil.

Parameter	Mackerel Oil	Sardine Oil	Sand Smelt Oil
Crude lipids	17.11 ± 0.10 ^a^	12.70 ± 0.28 ^b^	6.44 ± 0.14 ^c^
Specific gravity (SG)	0.912 ± 0.002 ^b^	0.947 ± 0.022 ^a^	0.928 ± 0.003 ^ab^
Refractive Index (RI)	1.479 ± 0.0021 ^a^	1.480 ± 0.01 ^a^	1.470 ± 0.001 ^a^
Flow time (sec.)	5.70 ± 0.01 ^b^	6.79 ± 0.01 ^a^	6.15 ± 0.06 ^ab^
Total carotenoids (µg/g)	128.34 ± 3.78 ^a^	98.12 ± 1.56 ^c^	105.26 ± 2.18 ^b^
Acid value (AV)	2.80 ± 0.07 ^a^	2.97 ± 0.05 ^a^	2.78 ± 0.01 ^a^
Peroxid value (PV)	2.67 ± 0.03 ^a^	2.67 ± 0.11 ^a^	2.28 ± 0.04 ^b^
Iodine value (IV)	182.89 ± 3.01 ^b^	194.33 ± 0.58 ^a^	109.67 ± 1.53 ^c^

Means within each row designated with the same letter are not significantly different at 0.05 level of probability, The same letter (superscripts) in each row are not significantly different at *p* ≤ 0.05, according to the Dencans test.

**Table 2:** Fatty acid composition (%) of investigated oil samples.

Compounds	Mackerel Oil	Sardine Oil	Sand Smelt Oil
Capric acid 10:0	0.02	Nd	0.025
Myristic acid 14:0	13.18	Nd	8.9
Palmitic acid 16:0	Nd	Nd	Nd
Palmitoleic acid 16:1	14.12	Nd	11.9
Stearic 18:0	7.04	7.16	11.82
Oleic 18:1	11.65	Nd	30.59
Linoleic 18:2	3.71	5.69	21.69
Linolenic 18:3	1.29	1.68	1.19
Gondoic acid 20:1	3.36	4.04	5.3
Arachidonic 20:4	3.97	4.55	1.09
Eicosapentaenoic acid (EPA) 20:5	18.63	23.27	1.7
Docosahexaenoic acid (DHA) 22:6	15.49	16.27	5.4
Total saturated fatty acids (SFA)	20.24	7.16	20.80
Total unsaturated (UnSFA)	72.22	55.5	78.86
Total monounsaturated fatty acids (MUFA)	29.13	4.04	47.79
Total polyunsaturated fatty acids (PUFA)	43.09	51.46	31.07
SFA/UnSFA	0.29	0.12	0.26

Nd: not detected; results are expressed as percentage of the total fatty acids.

**Table 3:** Physical properties of encapsulated fish oils.

Sample	Moisture %	Encapsulation Efficiency %	Flowability
WP + SSO	2.04 ± 0.09 ^c^	64.71 ± 0.26 ^c^	Fair
WP + MAO	1.93 ± 0.02 ^c^	71.16 ± 0.16 ^a^	Fair
WP + SRO	2.03 ± 0.07 ^c^	68.61 ± 0.24 ^b^	Fair
AG + SSO	3.45 ± 0.02 ^a^	47.06 ± 0.16 ^g^	Passable
AG + MAO	3.21 ± 0.08 ^a^	60.63 ± 0.33 ^d^	Passable
AG + SRO	3.51 ± 0.01 ^a^	56.98 ± 0.11 ^e^	Passable
MD + SSO	2.88 ± 0.02 ^b^	45.55 ± 0.10 ^h^	Poor
MD + MAO	2.49 ± 0.01 ^b^	57.25 ± 0.16 ^e^	Poor
MD + SRO	2.83 ± 0.05 ^b^	50.57 ± 0.51 ^f^	Poor

SSO = sand smelt oil; MAO = mackerel wastes oil; SRO = sardine wastes oil; MD = maltodextrin; WP = whey protein; AG = gum Arabic. The same letter (superscripts) in each column are not significantly different at *p* ≤ 0.05, according to the Dencans test.

**Table 4:** Flow properties of encapsulated fish oils.

Sample	Bulk Density (pB)	Tapped Density (pT)	Carr’s Index	Hausner Ratio	Flowability
WP + SSO	0.26 ± 0.05 ^ab^	0.32 ± 0.02 ^ab^	17.51 ± 1.18 ^ef^	1.21 ± 0.01 ^ef^	Fair
WP + MAO	0.22 ± 0.02 ^e^	0.27 ± 0.03 ^c^	16.24 ± 0.74 ^f^	1.19 ± 0.01 ^f^	Fair
WP + SRO	0.28 ± 0.05 ^a^	0.34 ± 0.01 ^ab^	18.64 ± 1.23 ^e^	1.23 ± 0.02 ^e^	Fair
AG + SSO	0.27 ± 0.05 ^ab^	0.35 ± 0.01 ^a^	23.08 ± 0.39 ^cd^	1.29 ± 0.06 ^c^	Passable
AG + MAO	0.26 ± 0.04 ^ab^	0.34 ± 0.02 ^ab^	21.77 ± 0.95 ^d^	1.27 ± 0.03 ^d^	Passable
AG+ SRO	0.26 ± 0.01 ^abc^	0.34 ± 0.02 ^ab^	23.89 ± 0.64 ^c^	1.32 ± 0.01 ^c^	Passable
MD + SSO	0.23 ± 0.15 ^de^	0.31 ± 0.02 ^b^	27.66 ± 0.43 ^b^	1.38 ± 0.07 ^b^	Poor
MD + MAO	0.24 ± 0.01 ^cde^	0.33 ± 0.01 ^ab^	26.51 ± 0.73 ^b^	1.36 ± 0.01 ^b^	Poor
MD + SRO	0.25 ± 0.02 ^bcd^	0.35 ± 0.02 ^a^	30.19 ± 0.42 ^a^	1.43 ± 0.01 ^a^	Poor

SSO = sand smelt oil; MAO = mackerel wastes oil; SRO = sardine wastes oil; MD = maltodextrin; WP = whey protein; AG = gum Arabic, The same letter (superscripts) in each column are not significantly different at *p* ≤ 0.05, according to the Dencans test.

**Table 5:** The ranges of flowability according to Carr’s index and Hausner ratio.

Flowability	Carr’s Index	Hausner Ratio
Excellent	<10	1.00–1.11
Good	11–15	1.12–1.18
Fair	16–20	1.19–1.25
Passable	21–25	1.26–1.34
Poor	26–31	1.35–1.45
Very poor	32–37	1.46–1.59
Awful	>38	>1.60

## Sample Availability

Samples of the compounds are available from the authors.
